# Neuroinflammatory Gene Expression Analysis Reveals Pathways of Interest as Potential Targets to Improve the Recording Performance of Intracortical Microelectrodes

**DOI:** 10.3390/cells11152348

**Published:** 2022-07-30

**Authors:** Sydney Song, Brianna Regan, Evon S. Ereifej, E. Ricky Chan, Jeffrey R. Capadona

**Affiliations:** 1 Department of Biomedical Engineering, Case Western Reserve University, 2071 Martin Luther King Jr. Drive, Cleveland, OH 44106, USA; sss176@case.edu (S.S.); eereifej@gmail.com (E.S.E.); 2 Advanced Platform Technology Center, Louis Stokes Cleveland Veterans Affairs Medical Center, Cleveland, OH 44106, USA; 3 Veteran Affairs Ann Arbor Healthcare System, Ann Arbor, MI 48105, USA; reganbri@umich.edu; 4 Department of Biomedical Engineering, University of Michigan, Ann Arbor, MI 48109, USA; 5 Department of Neurology, University of Michigan, Ann Arbor, MI 48109, USA; 6 Institute for Computational Biology, Case Western Reserve University, Cleveland, OH 44106, USA; erc6@case.edu

**Keywords:** microelectrode, inflammation, cluster of differentiation 14, Toll-like receptors, cytokine, complement, extracellular matrix

## Abstract

Intracortical microelectrodes are a critical component of brain-machine interface (BMI) systems. The recording performance of intracortical microelectrodes used for both basic neuroscience research and clinical applications of BMIs decreases over time, limiting the utility of the devices. The neuroinflammatory response to the microelectrode has been identified as a significant contributing factor to its performance. Traditionally, pathological assessment has been limited to a dozen or so known neuroinflammatory proteins, and only a few groups have begun to explore changes in gene expression following microelectrode implantation. Our initial characterization of gene expression profiles of the neuroinflammatory response to mice implanted with non-functional intracortical probes revealed many upregulated genes that could inform future therapeutic targets. Emphasis was placed on the most significant gene expression changes and genes involved in multiple innate immune sets, including *Cd14*, *C3*, *Itgam*, and *Irak4.* In previous studies, inhibition of Cluster of Differentiation 14 (*Cd14*) improved microelectrode performance for up to two weeks after electrode implantation, suggesting CD14 can be explored as a potential therapeutic target. However, all measures of improvements in signal quality and electrode performance lost statistical significance after two weeks. Therefore, the current study investigated the expression of genes in the neuroinflammatory pathway at the tissue-microelectrode interface in *Cd14*^−/−^ mice to understand better how *Cd14* inhibition was connected to temporary improvements in recording quality over the initial 2-weeks post-surgery, allowing for the identification of potential co-therapeutic targets that may work synergistically with or after CD14 inhibition to improve microelectrode performance.

## 1. Introduction

Intracortical microelectrodes were initially developed as a tool to interpret the functional circuitry of the brain because of their ability to allow neuronal communication for analysis and functional outputs [1]. When implanted, intracortical microelectrodes can record the action potentials of single neurons or a group of neurons. This allows for advancing brain-machine interface (BMI) technology, which improves clinical applications [2,3,4,5,6]. BMIs aim to treat individuals suffering from neurological disorders and spinal cord injuries [7]. Clinical studies using chronically implanted electrodes for BMIs have enabled individuals to move a computer cursor in three dimensions [8,9], control a robotic arm [10,11,12], or restore function to their disabled limb [13].

Unfortunately, implanted microelectrode devices fail prematurely. Within months to years after implantation, the quantity and quality of signals obtained from intracortical microelectrodes decrease, as measured by metrics such as the number of channels capable of recording single-unit neuronal activity or signal-to-noise ratio [14]. Without quality signals, the clinical usefulness of the microelectrodes to patients who may benefit from the recording abilities of these devices is minimal.

Many labs have sought to prolong the lifespan of the intracortical microelectrodes by exploring many mechanisms to promote a reduction of the inflammatory response, including (but not limited to): minimizing the trauma associated with device implantation [15,16], minimizing the device/tissue stiffness mismatch [17,18,19,20,21,22,23,24,25], better understanding the effect of device sterility [26,27], reducing oxidative stress/damage [19,28,29,30,31,32,33,34], and mimicking the nano-architecture of the natural extracellular matrix [35]. The complexity of understanding so many different approaches to mitigate the self-perpetuating inflammatory response to intracortical microelectrodes has led us to focus our investigations on understanding the role of specific aspects of the inflammatory response.

To that end, we are interested in understanding the role of innate immune pathways and changes in the gene expression of inflammation-associated molecules after microelectrode implantation. Therefore, we recently characterized the gene expression profiles of the neuroinflammatory response to mice acutely implanted with non-functional intracortical probes [36]. Differential gene expression analysis identified that the most significant changes in gene expression occur 24-h post-surgery and in genes involved in multiple innate immune sets, including *Cd14*, *C3*, *Itgam*, and *Irak4*. While *Cd14* showed upregulation throughout the 2-week study, it showed the most significant upregulation (~5–6 log2foldchange) in the initial 24-h post-implantation—indicating that downstream events following Cd14 expression may be an indicator of microelectrode performance. Due to its essential role in the innate immune system as a pattern recognition molecule that helps to initiate an innate immune response, we had already been interested in *Cd14* (Cluster of Differentiation 14) and have been investigating its role in microelectrode performance before the gene expression study, including exploring its potential as a therapeutic target [18,37].

In response to injury or infection, the activation of first-responder microglial and macrophage cells is initiated through a signaling cascade that begins with cell surface receptors. These receptors recognize plasma proteins and damage-associated molecular patterns (DAMPs) in the damaged tissue or adsorbed on the surface of the implanted microelectrodes. CD14 is a primary receptor in the inflammatory response to implanted intracortical microelectrodes. CD14 is a co-receptor for many Toll-like receptor (TLR) subtypes, including TLR2 and TLR4. CD14 is expressed in many innate immune cells such as microglia, macrophages, dendritic cells, and to a lesser extent, nonimmune cells in the brain such as astrocytes and neurons [38,39,40,41], with a primary role of recognition of DAMPs; again suggesting that that downstream events following Cd14 activation may be an indicator of microelectrode performance.

We have investigated the TLR/CD14 pathway’s role in chronic recording performance and reduce inflammation around brain-electrode interfaces. Specifically, complete inhibition of *Cd14* using a *Cd14*^−/−^ mouse model improved recording during acute but not chronic time points [37,42]. Since CD14 is involved in the initial recognition and response to intracortical microelectrode implantation, eliciting a complex neuroinflammatory response, it is essential to better understand how inhibition of *Cd14* through deletion resulted in initial improvements in recording performance to maintain chronic neural recordings.

Therefore, the goal of this study was to develop a gene expression-level understanding of how *Cd14* inhibition was connected to temporary improvements in recording quality over the initial two weeks post-surgery and identify other genes in the inflammatory pathway that may be contributing to microelectrode failure and identify potential co-therapeutic targets with CD14 inhibition. Here, we evaluated the gene expression profiles of 791 genes isolated from the tissue around intracortical microelectrodes implanted in *Cd14*^−/−^ mice. We compared gene expression profiles to genotype-matched naïve, non-surgical (NSCTR) mice.

## 2. Materials and Methods

### 2.1. Animals

All animal care, handling, and procedures were performed in compliance with a protocol approved by the Institutional Animal Care and Use Committee (IACUC) at Case Western Reserve University. A total of 25 male *Cd14*^−/−^ (Jackson Laboratory Strain #003724) mice were used in this study. We have not found evidence of *Cd14* being linked to sex-specific neuroinflammatory responses and thus started with male mice. Future studies will explore the potential for sex-specific effects of *Cd14* inhibition. All mice were obtained from Jackson laboratory between 7–10 weeks of age. Animals were housed in ~3–5 per cage for 1–4 weeks before surgery. All animal handling was conducted in a class II sterile hood using microisolator techniques. Animals used in this study were randomly divided into endpoint groups (6-h, 24-h, 72-h, and two weeks), with additional animals used as NSCTR. Each group had five animals. NSCTR animals were all male, age-matched, and had no pre-, post-, or surgical procedures. After surgery, all animals were singly housed to prevent physical interactions that may displace implanted electrodes. Genotyping was confirmed after gene expression analysis was performed.

### 2.2. Microelectrodes

Non-functional, Michigan-style silicon shank probes (provided by Pancrazio Lab at the University of Texas at Dallas) were used in this study (15 µm thick, 123 µm at its widest part, and 2 mm long). All probes were washed by soaking in 95% ethanol solution three times, five minutes each, and sterilized with cold ethylene oxide gas, as previously described [26,35,43]. Non-functional probes were utilized in this study for consistency with our previous study using wild-type mice. Unfortunately, non-functional probes limited our ability to link our findings in the current study directly to device performance [36].

### 2.3. Surgical Procedure

Surgical procedures were performed following established laboratory protocols [36]. Briefly, mice were sedated with isoflurane; 3% in 1.0 L/min O_2_ for induction and ~2% during surgery. The surgical site was shaved. The animals were placed on a stereotaxic frame and given a single dose of 0.2 mL of 0.25% Marcaine subcutaneously (SQ) around the surgical site as a topical anesthetic. Next, the skin was sterilized using betadine and isopropanol dipped swaps, then incised at midline to expose the skull, and a hydrogen peroxide swab cleaned off tissue. A 0.45 mm dental drill was used for the craniotomy, pulsing the drill to allow heat to dissipate; a total of 4 holes were drilled at 1.5 mm lateral and 1.0 mm anterior and posterior to the bregma, pulsing the drill to allow heat to dissipate [15]. Nonfunctional probes were inserted manually perpendicular to the surface of the brain into each hole, taking care to avoid large visible vasculature. We chose manual insertion methods to be consistent with previous studies in our lab [36]. The same surgeon performed all implantations to mitigate the surgery variability between animals. Probes contain a section of the tab that is wider than the drilled hole, such that the tab will stay above the skull after implantation, ensuring that the depth of the microelectrode will be consistent between implant sites. Nonfunctional probes are held by fine tip forceps at the tab and slowly implanted into the cortex by hand (at an estimated rate of ~2–3 mm/s) until the 2 mm long shank was implanted. The craniotomy hole was sealed with Kwik-Sil, and dental cement (Flow-It) was used to tether the silicon probe to the skull. The incision was then closed with a 5–0 monofilament polypropylene suture. Post-operative pain management included daily Meloxicam (2 mg/kg, SQ) and Buprenorphine (0.05 mg/kg, SQ) for 3 days post-surgery. Since our veterinary care requires that we commonly use Meloxicam, we chose to be consistent with our prior and current practices and use it here, despite the potential to influence the more acute time points.

### 2.4. Tissue Extraction

All animals were anesthetized with a ketamine-xylazine cocktail (100 mg/Kg and 10 mg/Kg, respectively) and euthanized via cardiac perfusions with cold 1× phosphate-buffered saline (PBS). Brains were immediately extracted, and probes (if implanted) were explanted. Perfusion and explanation were done quickly to prevent excessive degradation of RNA. Brain tissue was flash frozen in optimal cutting temperature compound (OCT) on dry ice and stored at −80 °C until further processing. Using a cryostat, the cortical brain tissue surrounding the neural probes was sectioned into 150 μm thick frozen slices. A biopsy punch (1 mm diameter) was used to excise the tissue of the frozen tissue slices immediately. The resulting tissue samples 500 μm radii from the implant site. Six slices were collected per animal for a depth of 900 μm into the cortical tissue. Tissue collection started at ~150 μm depth, continuing down the length of the device, spanning most of the cortex.

### 2.5. RNA Isolation

Extracted brain tissue was homogenized by placing collected samples directly into 2.0 mL homogenization microtubes prefilled with 1.5 mm zirconium beads (Benchmark scientific D1032-15) and 1 mL Qiazol (RNA extraction lysate) [36]. The microtubes were then loaded onto a Bead Bug Homogenizer (Benchmark Scientific D1030) and shaken at 4000 rpm for 1 min.

The RNA was extracted and purified from homogenized tissue using RNeasy^®^ Plus Universal Mini Kit (Qiagen 73404) at the Gene Expression and Genotyping Facility at Case Western Reserve University. RNA quality and quantity were determined using Nanodrop. Samples with low concentration were concentrated with Speedvac. Isolated RNA was stored at −80 °C for up to two months. Samples were shipped overnight on dry ice to NanoString Technologies (Seattle, WA, USA) for further quality control and quantification.

### 2.6. Gene Expression Assay

Gene expression is determined by counting individual genes using a digital color barcode technology developed by NanoString Technologies (Seattle, WA, USA) [43]. For each sample, 100 ng of RNA was hybridized with a codeset containing capture probes and reporter probes genes of interest. Here, we utilized a codeset containing 791 genes; 771 were from the nCounter^®^ Mouse Neuroinflammation Panel, which included 13 housekeeping genes, and an additional 20 custom genes of interest (Table 1). Negative controls and positive controls were spiked in. Samples were incubated at 65 °C for 16 h, then loaded onto cartridges and processed with nCounter^®^ Max Analyzer. Measurements were taken at 280 Field-of-View per sample, and the relative number of each gene was determined from absolute counts of fluorescent barcode reporters using the nCounter^®^ MAX Analyzer.

### 2.7. Data Visualization and Statistical Analysis

#### 2.7.1. Normalization

Normalization was performed with the software nSolver (v 4.0) and Advanced Analysis Plugin of nSolver (v 2.0.115), developed by Nanostring Technologies [44,45,46]. Raw counts for each sample were normalized to both the spiked-in positive controls and housekeeping gene controls. Ten housekeeping genes were used for normalization. Genes with counts below 25 in 85% of the samples were excluded from the analysis.

#### 2.7.2. Heatmap and Principal Component Analysis

To visualize the overall variation in gene expression, heatmap and principal component analysis [47,48] was performed on the normalized and log2 transformed sample counts to help visualize the variation between samples using ClustVis [49].

#### 2.7.3. Comparison of Gene Expression at Each Post-Surgical Time Point to Naïve Non-Surgical Control

To examine the change in gene expression after implantation, nSolver and Advanced Analysis Plugin of nSolver, developed by Nanostring Technologies, were used to calculate the ratio between each time point (6-h, 24-h, 72-h, and two weeks) and the naïve non-surgical control [50]. The ratio was then plotted on a log2 scale (hereafter referred to as log2foldchange). The standard error of the mean between each time point and non-surgical control was calculated and plotted for each pair. Unpaired T-test with Benjamini-Yekutieli False-Discovery-Rate Correction is used to determine statistical significance. Significance is set at P_adj_ < 0.05 [36].

Based on the analysis above, genes with altered expression at threshold log2foldchange > 1 or <−1, (or 2-fold increase or decrease in expression), P_adj_ < 0.05, at overlapping time points, are counted and visualized with a Venn diagram. Volcano plot and bar graph [51,52] of altered expression of specific genes are generated using Matlab (R2021B, MathWorks, Natick, MA, USA).

## 3. Results

### 3.1. Overall Gene Expression

We have shown that complete inhibition of *Cd14* resulted in temporary improvements in microelectrode performance [37,42]. Therefore, the goal of this study was to develop a gene expression-level understanding of the progression of the neuroinflammatory response to microelectrode implantation, to understand how inhibition of *Cd14* expression improved microelectrode performance, and to identify potential therapeutic targets that can be inhibited alone or synergistically with *Cd14* inhibition to improve microelectrode performance.

**Figure 1:**
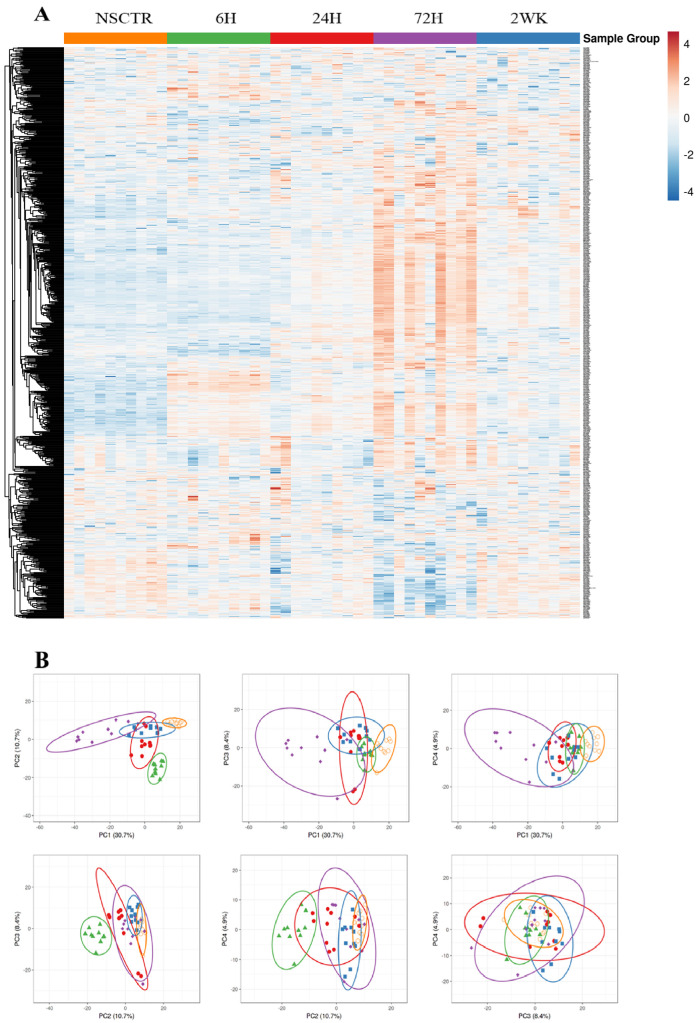
Heat map and principal component analysis. (**A**) Heatmap of gene expression after normalization and log2 transformation. (**B**) principal Component Analysis of normalized log2 transformed data. PC1—PC4 is displayed, and sample groups are marked. The first 4 Principal Component axes account for a total of 55.7% variation in the data. Specifically, PC1 accounts for 30.7% of the variation in data, PC2 accounts for 10.7% of the variation in data, PC3 accounts for 8.4% of the variation in data, and PC4 accounts for 4.9% of the variation in data. New samples are predicted to fall within the elliptical with a probability of 0.95. Orange (open circles) = NSCTR; Green (triangles) = 6-h; Red (circles) = 24-h; Purple (diamonds) = 72-h; and Blue (squares) = 2-week.

Here, we evaluated the gene expression profiles of 791 genes isolated from tissue surrounding intracortical microelectrodes implanted in *Cd14*^−/−^ mice. We compared gene expression profiles to genotype-matched naïve, non-surgical control (NSCTR) mice. We began our analysis by generating a heatmap to visualize changes in gene expression with respect to time and variation between samples within a set using ClustVis [49]. To account for variability within the same animal, we used tissue adjacent to two of the four implant sites per animal for five animals (and ten implant sites) per condition/time point (Figure 1A). Visual inspection suggests that gene expression patterns within animal sets for a given time point are more consistent than across time points with some variation within time point groupings.

Therefore, we next performed Principal Component Analysis (PCA) to further visualize the overall gene expression variation on normalized log2 transformed sample counts (Figure 1B). The first four axes of principal component analysis are displayed. For the first four principal axes (of 791 axes), PC 1–4 has a combined score of 54.7% (or accounts for 54.7% of the variation in data). PC 1 score is 30.7%, while PC2, PC3, and PC4 scores are 10.7%, 8.4%, and 4.9%, respectively. The elliptical around each group shows a prediction space, where any new sample of the same group is predicted to fall within the elliptical with a probability of 0.95. The larger the elliptical, the greater gene expression variation within a sample group. Both the heat maps and PCA demonstrated that pre-surgery gene expression of the inflammatory pathway is similar across samples. The projection associated with gene expression at a 6-h post-surgical time point on PC2 decreases while the variation increases compared to the NSCTR. The projection associated with gene expression at the 24-h post-surgical time point decreases on the PC1 axis and continues to increase in variation compared to NSCTR. At 72-h post-surgery, gene expression showed the greatest variation, and the associated projections decreased further on PC1 compared to NSCTR. By two weeks post-surgery, the projections of gene expression are located close to that of NSCTR compared to 6–72-h post-surgical time points. However, expression at 2-week time points still showed increased variation compared to NSCTR.

**Figure 2:**
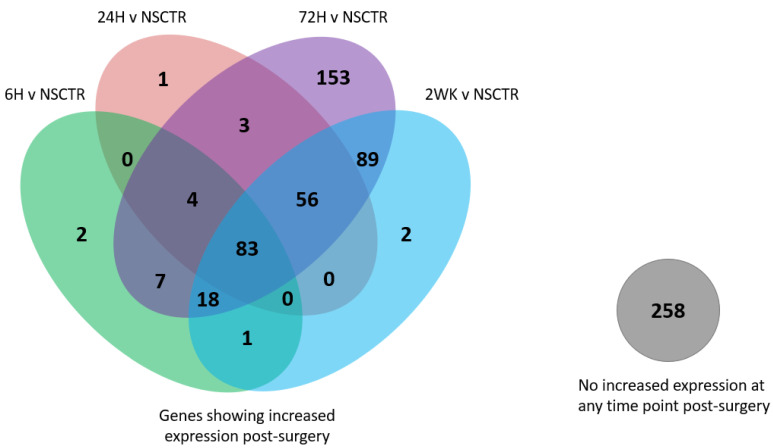
Venn diagram of the number of genes showing altered expression post-surgery compared to Non-Surgical Control (NSCTR). Only genes above the expression threshold of 25 counts in over 85% of the samples are included. (log2foldchange > 1 or <−1, P_adj_ < 0.05). Overlapping points on the diagram (blended color) indicate the same genes demonstrating altered expression across both time points.

We next created a Venn diagram to display the number of genes showing altered expression post-surgery compared to NSCTR mice (Figure 2). Only genes above the expression threshold of 25 counts in over 85% of the samples are included. Overall, two-hundred-and-fifty-eight genes did not show changes in the expression above the threshold (log2foldchange > 1 or <−1, or 2-fold increase or decrease in expression, P_adj_ < 0.05) compared to NSCTR mice at any post-surgical time point, and only seven genes demonstrated a reduced expression (not shown in the figure). However, eighty-three genes showed changes in expression at all post-surgical time points compared to control. Genes showing increased expression above the threshold at early post-surgerical time points, 6-h, and 24-h post-surgery, tended to continue expression above the threshold until 72-h and 2-week post-surgical time points. Two genes showed changes in expression at only 6-h post-surgery. One gene showed changes in expression at only 24-h post-surgery. Four genes maintained increased expression from 6-h until 72-h post-surgery. Eighty-three genes maintained increased expression from 6-h to 2-weeks post-surgery. Fifty-six genes showed increased expression from 24-h to 2-weeks post-surgery. Additionally, one-hundred-and-fifty-three genes showed changes in expression at only 72-h post-surgery, two genes showed changes in expression at only 2-weeks post-surgery, and eighty-nine genes showed increased expression beginning at 72-h post-surgery and continued until 2-week post-surgical time point.

Most of the genes showed an increase in expression after surgery, which was expected when focusing on neuroinflammatory genes. The highest upregulation in gene expression occurs at the 72-h time point, as indicated by several genes upregulated at 72-h post-surgery (Figure 2). Compared to WT mice implanted with microelectrodes, where the highest gene expression level is at 24-h [36], delayed upregulation of proinflammatory genes may help improve microelectrode performance initially—indicating a possible reason for initial but not sustained improvements in microelectrode recording performance in *Cd14*^−/−^ mice. Additionally, at 72-h post-surgery, the variability in gene expression within both *Cd14*^−/−^ and WT mice reaches the maximum, corresponding to a transitional period in wound healing [53]. The cellular responses transition from predominantly neutrophils to predominantly macrophages [54]. Lempka et al. also showed that impedance transitions from low to high between days 3–5 post-implantation of deep brain stimulating electrodes [55]. Therefore, the neuroinflammatory response at 72-h post-surgery may correlate and predict long-term microelectrode variability and performance, suggesting potential interest for future interventional research.

### 3.2. The Complement Pathway

The complement system is a component of the innate immune system. The complement system comprises both circulating and membrane-bound proteins and proteases and can opsonize foreign substances for clearance and destruction by phagocytes, such as microglia and macrophages [56]. We and others have previously shown that the complement system is upregulated when an intracortical microelectrode is implanted in mice [36,51].

**Figure 3:**
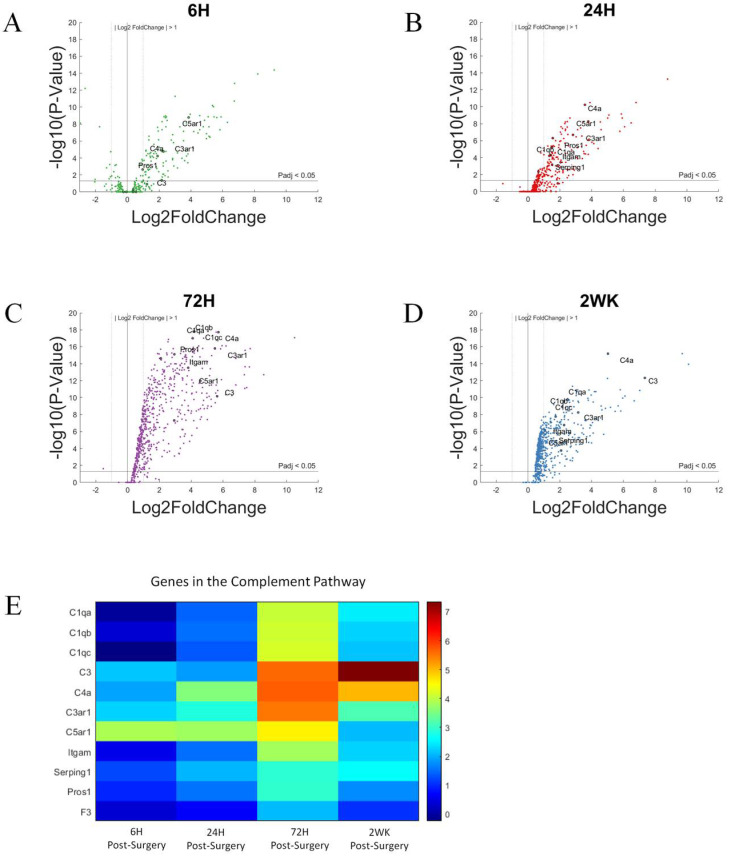
Differential expression of gene set involved in the complement pathway compared to NSCTR mice: (**A**–**D**) volcano plot with genes in the complement pathway shown in black circles. Top 10 genes by differential expression level and P_adj_ < 0.05 are labeled. Each time point post-surgery is on a separate volcano plot. (**A**) =6-h, (**B**) =24-h, (**C**) =72-h, and (**D**) =2-weeks. Color in (**A**–**D**) corresponds to time post-surgery color coding in other figures. (**E**) heatmap showing differential expressions of genes of the complement system at each time point post-surgery compared to NSCTR.

Here, we generated volcano plots for each of the time points investigated. The volcano plots visualize increases in the gene expression for all the genes we examined within a given time point compared to NSCTR mice (Figure 3A–D). Here, we focus on the genes that participate in the complement cascade: *C1qa*, *C1qb*, *C1qc*, *C3*, *C4a*, *C6*, *C3ar1*, *C5ar1*, *Itgam*, *Cd19*, *Serping1*, *Pros1*, and *F3*. These genes are labeled in the volcano plot, if P_adj_ < 0.05 and log2foldchange > 1 or <−1 (i.e., 2-fold increase or decrease in expression). Furthermore, due to the large number of gene in this grouping, only the top 10 genes with the largest log2foldchange at each time point within the group are labeled. At 6-h post-surgery, *C4a*, *C3*, *C3ar1* and *C5ar1* increased gene expression compared to non-surgical control (Figure 3A). At later time points, 24-h (Figure 3B), 72-h (Figure 3C), and 2-week (Figure 3D) post-surgery, most genes of the complement system showed increased gene expression and remained elevated throughout the first two weeks post-surgery. The relative increase in gene expression levels for each of these genes associated with the complement cascade are more readily depicted in heatmaps (Figure 3E).

**Figure 4:**
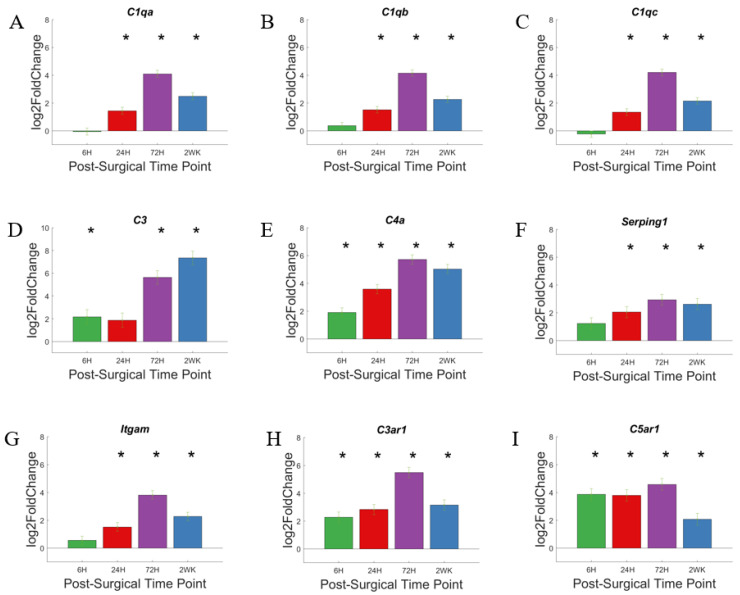
Differential expression of specific genes involved in the complement pathway compared to NSCTR mice: Top differentially expressed genes for the complement pathway displayed as bar graphs of individual genes as a function of time post-surgery (**A**–**I**). For each time point, gene expression levels are compared to the NSCTR mice. Error bars indicate the standard error of the mean between NSCTR and each time point. Asterisks indicate that P_adj_ < 0.05. Note (**D**), which depicts the upregulation of *C3*, has a y-axis log2foldchange scale of −1 to 10, because of its high upregulation.

Genes with the highest differential expression include *C3*, *C4a*, *C3ar1*, and *C5ar1*, which code genes for the amplification of the complement system (Figure 3E and Figure 4). *C1qa*, *C1qb*, and *C1qc* encode the protein C1q, which is a component of the C1 complex, which in turn initiates the activation of complement cascade via the Classic Pathway [57]. All three genes show a similar trend in their expression level throughout this study (Figure 4A–C): the expression levels increase after 24-h post-surgery, reaching a maximum at 72-h post-surgery, and remain elevated at 2-weeks post-surgery. *C3* encodes complement factor 3, which marks both an activation and an amplification step in the complement cascade, as well as acting as a signaling molecule [56]. *C3* (Figure 4D) shows a gradual upregulation over 2 weeks. *C4a* codes for a portion of complement factor 4, C4a; C4a, in turn, is a product of complement activation and acts as a signaling molecule to recruit other immune cells [56]. Both *C3* (Figure 4D) and *C4a* (Figure 4E) show a gradual upregulation over the first 72-h time point, and while remaining highly expressed, decrease slightly by the 2-week time point. The inhibitor of the C1 complex, *Serping1* (Figure 4F), follows the same trend as *C4a*, although more modest. *Itgam*, a subunit of C3 receptor, also showed gradually increased expression, not statistically significant at 6-h, but significant by 24-h, reaching maximum at 72-h post-surgery, and remains elevated at 2-week post-surgical time point (Figure 4G). *C3ar1* (Figure 4H) and *C5ar1* (Figure 4I) show increased expression relatively early and remain elevated. Together, the increased expression of the soluble proteases of the complement system, as well as its receptors, suggest that the complement system may be involved in the response to the implanted microelectrode.

The complement pathway can be initiated via the classical pathway, lectin-binding pathway, or alternative pathway. All three pathways converge on the amplification step of C3 [58,59]. While most of the members of the complement system begin to show less upregulation by 2-weeks post-surgery, C3 continues to show an increase in upregulation of gene expression, increasing with each time point evaluated here. Note that C3 itself can initiate the activation of complement cascade via the alternative pathway. Therefore, C3′s steadily increased upregulation may drive complement activity beyond the 2-week course of our study.

The complement system may be involved in the response to biomaterials [60,61,62]. Biomaterials surface adsorption of IgG or hydrophobic interaction with C3 may lead to the activation of the complement cascade. Cells of the innate immune system can recognize adsorbed IgG or C3 through cell surface receptors, activating the inflammatory cascade through the release of cytokines and chemokines—further recruiting additional immune cells to the implantation site [63,64,65]. While the complement system has been implicated in the foreign body response to devices used for extracorporeal circulation [66,67,68], few studies have begun to investigate the role of complement system in foreign body response against intracortical microelectrode implant [36,51]. The observation that the genes associated with the complement system are upregulated throughout the duration of this study does not correspond with the observation that intracortical microelectrode recording performance initially improves, then subsides to match wild-type mice in *Cd14*^−/−^ mice [42]. Therefore, it is unlikely that the complement pathway is contributing to the temporal changes in recording performance in *Cd14*^−/−^ mice, unless there is a threshold effect, as C3 expression continues to rise with time post-surgery (Figure 4D). However, the high upregulation of many members of the complement system in both *Cd14*^−/−^ mice and WT mice [36] suggests it may play an important role in inflammatory response against implanted microelectrodes, and can be a potential target to improve microelectrode performance, either alone or as a co-therapeutic target with CD14. It is also important to point out that C3 has also been implicated as a marker of astrocyte maturation, and therefore we cannot overlook the possibility that C3 expression in this system may have downstream effects on microelectrode performance and the neuroinflammatory response, even if the timing of C3 expression seen here do not correlate with recording performance over the initial 2 weeks following microelectrode performance.

### 3.3. Pattern Recognition Receptors

Pattern recognition receptors (PRR) are part of the innate immune pathway that respond to evolutionarily conserved Pathogen Associated Molecular Patterns (PAMPs) and Damage Associated Molecular Patterns (DAMPs). The identification of PAMPs and DAMPs by PRRs indicates the presence of infection or injury, initiating the innate and adaptive immune responses [69]. PRRs can be broadly divided into membrane-bound or scavenger receptors [69,70,71,72,73]. The membrane-bound receptors include Toll-like receptors (TLRs) and C-Lectin receptors (CLRs), and cytoplasmic class: Nod-like receptors (NLRs), Aim2-like receptors (ALRS), and Rig-I like receptors (RLRs). TLRs will be discussed in depth in the next section.

**Figure 5:**
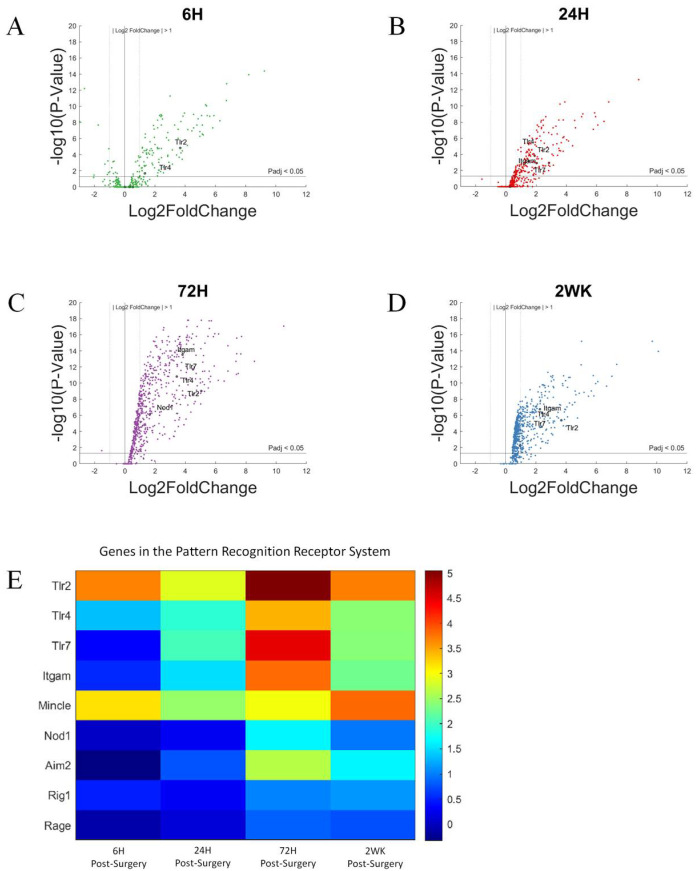
Differential expression of gene set involved in the pattern recognition system compared to NSCTR mice: (**A**) volcano plot with genes in the PRR pathway are shown in black. Genes in the pattern recognition system with P_adj_ < 0.05 and log2foldchange > 1 or <−1 are labeled. Each time point post-surgery is on a separate volcano plot. (**A**) =6-h, (**B**) =24-h, (**C**) =72-h, and (**D**) =2-weeks. Color in (**A**–**D**) corresponds to time post-surgery color coding in other figures. (**E**) heatmap showing differential expressions of genes of the chemokine system at each time point post-surgery compared to NSCTR.

Using the same volcano plots generated for all genes investigated for this study, we here (Figure 5A–D) labeled genes associated with the pattern recognition receptors, including: *Tlr2*, *Tlr4*, *Tlr7*, *Itgam*, *Mincle*, *Nod1*, *Aim2*, *Rig1*, and *Rage*. The given genes were only indicated on the volcano plot if P_adj_ < 0.05 and log2foldchange > 1 or <−1 (i.e., 2-fold increase or decrease in expression). At 6-h post-surgery, *Tlr2*, *Tlr4*, and *Mincle* increased gene expression compared to non-surgical control. These genes remained elevated throughout all time points up to 2-weeks post-surgery. By 24-h post-surgery, *Itgam* expression increased to be included in the PRR associated gene expression. *Itgam* remained upregulated at each of the later time points investigated in this study (Figure 5B–D). At 72-h post-surgery, *Nod1* and *Tlr7* became upregulated, joining *Tlr2*, *Tlr4*, *Mincle*, and *Itgam*. By 2-weeks post-surgery, *Nod1* expression is reduced to no longer be significantly upregulated compared to controls, while the other 5 genes remain elevated compared to control animals.

**Figure 6:**
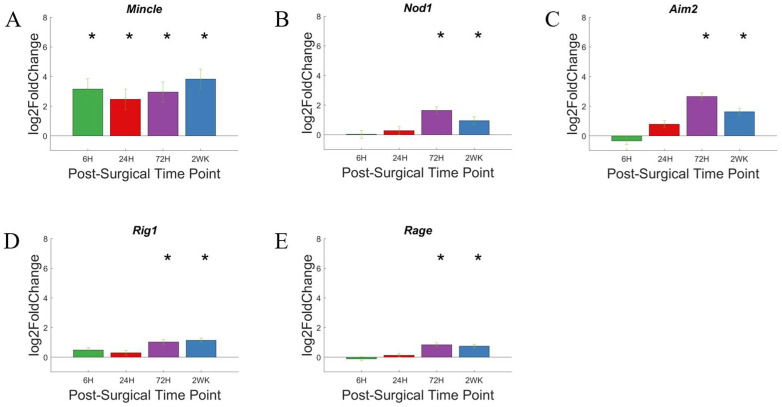
Differential expression of specific genes in the pattern recognition receptor family compared to NSCTR mice: All genes for the pattern recognition receptor except Toll-like receptors, which will be described in Figure 8. Gene set displayed as bar graphs of individual genes as a function of time post-surgery (**A**–**E**). For each time point, gene expression levels are compared to the NSCTR mice. Error bars indicate the standard error of the mean between NSCTR and each time point. Asterisks indicate that P_adj_ < 0.05.

The relative increase in gene expression levels for each of these genes for the pattern recognition receptors are more readily depicted in heatmaps (Figure 5E), with statistical significance more clearly depicted in bar graphs (Figure 6). *Tlr2*, *Tlr4*, *Tlr7*, and *Itgam* are discussed in the next section, Toll-Like Receptors.

To look closer at changes in individual genes over time, we created bar graphs to better visualize statistically relevant changes. In the bar graphs (Figure 6) created for individual genes, we can see that Nod1, Aim2, Rig1, and Rage does not show statistically significant upregulation until 72-h post-surgery and remain so at 2-weeks post-surgery. The extent of upregulation of each of these genes is relatively low, compared to other genes associated with the PRR pathway. For example, Nod1, Aim2, Rig1, and Rage reach a high log2foldchange of ~2, ~2.5, ~1.5, and ~1, respectively. Nod1, Aim2, and Rig1 are representative genes in the Nod-like receptors (NLRs), Aim2-like receptors (ALRs), and Rig−1 like receptors (RLRs) class of pattern recognition receptors, encoding for cytoplasmic proteins [69]. However, Rage encodes for a scavenger receptor. The delayed response of these genes suggest that they could be potential co-therapeutic targets together with CD14, which displays a rapid response and can be targeted for microelectrode performance at acute time points. [42]. Inhibition can be given sequentially, targeting CD14 during the acute phase of post-surgical implantation of microelectrodes, and later switch to targeting a slower upregulated pattern recognition receptors.

*Mincle*, on the other hand, showed upregulation at 6-h post-surgery, and maintained similar expression throughout the 2-week study. *Mincle* codes for a protein in the CLR class of pattern recognition receptors and has been implicated in neuroinflammation and injury in the central nervous system [42,74]. Mincle could be further explored as either a solo therapeutic target or a co-therapeutic target together with CD14.

### 3.4. Toll-Like Receptors and Associated Pathways

Toll-like receptors are a subset of pattern recognition receptors that are membrane-bound. Some of its members, such as TLR 2 and TLR4, are bound to plasma membrane, while others, TLR3, TLR9, are bound to endosome membrane [75]. Note that the mice used in this study were *Cd14*^−/−^, and CD14 is a co-receptor for TLR2 and TLR4. Previously, our lab has investigated the role of Toll-like receptors in the neuroinflammatory response to intracortical microelectrodes and the associated recording performance. In our previous studies, we concluded that while complete inhibition of TLR2 had no impact on tissue response to microelectrode, complete inhibition of TLR4 worsened tissue response [76].

**Figure 7:**
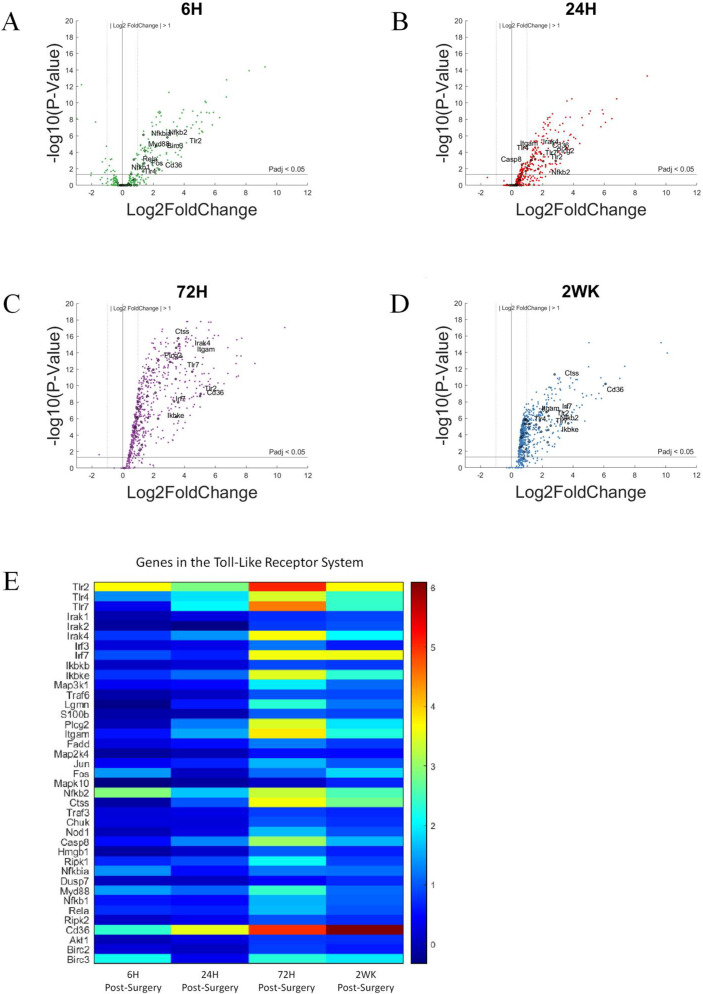
Differential expression of gene set involved in the Toll-like receptor pathway compared to NSCTR mice: (**A**) volcano plot with genes in the TLR group shown in black circles. Top 10 genes by differential expression level and P_adj_ < 0.05 are labeled. Each time point post-surgery is on a separate volcano plot. (**A**) =6-h, (**B**) =24-h, (**C**) =72-h, and (**D**) =2-weeks. Color in (**A**–**D**) corresponds to time post-surgery color coding in other figures. (**E**) heatmap showing differential expressions of genes of the TLR system at each time point post-surgery compared to NSCTR.

In the volcano plots (Figure 7A–D), genes associated with Toll-like receptor pathway are labelled if P_adj_ < 0.05 and log2foldchange > 1 or <−1 (i.e., 2-fold increase or decrease in expression). Furthermore, due to the large number of gene in this grouping, only the top 10 genes with the largest log2foldchange at each time point within the group are labeled. With a few exceptions such as *Tlr2*, *Nfk2*, and *Cd36*, the genes of the Toll-like receptor pathway are slow to increase in expression. Whereas there are fewer genes showing upregulation at 6-h post-surgery, and more genes showing upregulation in gene expression at 72-h post-surgery. This is different than the time course of expression for genes in the Toll-like receptor pathway in WT animals. Specifically, we have demonstrated that in WT mice implanted with intracortical microelectrodes, the genes in the Toll-like receptor pathway show an upregulated expression early on [36]. This distinction in the *Cd14*^−/−^ mice is most likely due to the lack of CD14 requiring a secondary mechanism to initiate the TLR-mediated neuroinflammatory response and could be directly linked to initial and short-lived improvements in recording performance in *Cd14*^−/−^ mice [42].

**Figure 8:**
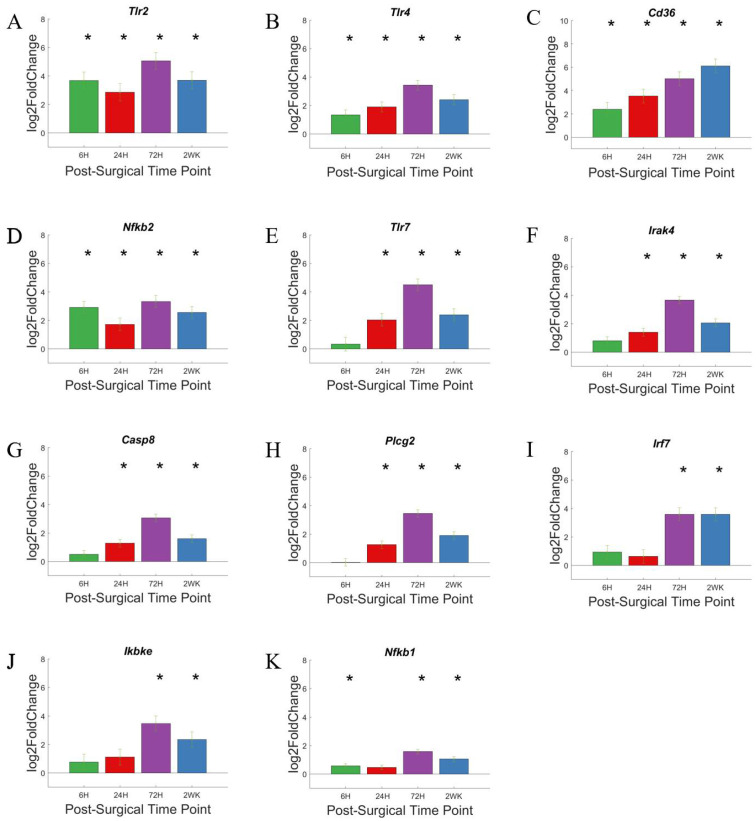
Differential expression of specific genes involved in the Toll-like receptor pathway compared to NSCTR mice: Bar graph of selected genes in the Toll-like Receptor’s pathway (**A**–**K**), alterations in expression are displayed as bar graphs of individual genes as a function of time post-surgery. For each time point, gene expression levels are compared to the NSCTR mice. Error bars indicate the standard error of the mean between NSCTR and each time point. Asterisks indicate that P_adj_ < 0.05. Note (**C**), depicting the upregulation of *Cd36*, has a y-axis log2foldchange scale of −1 to 10, because of its high upregulation.

Again, we created bar graphs to better visualize statistically relevant changes in gene expression as a function of time (Figure 8). Here, we see that the expression of *Tlr2*, *Tlr4*, *Cd36*, and *Nfkb2* (Figure 8A–D) all displayed elevated gene expression at all four time points investigated. However, each of these four genes displays different levels and a different pattern of activation. For example, *Tlr2* and *Nfkb2* are relatively consistent over time, with slight fluctuations both up and down. Alternatively, *Cd36* expression is the only gene in the TLR pathway that continues to increase with each subsequent time point that we evaluated. Therefore, the continuous increase in *Cd36* expression suggests that increasing expression could be related to delayed activation or downstream compensation resulting from the lack of CD14.

Of note, *Tlr7*, *Irak4*, *Casp8*, *Picg2*, *Irf7* and *Ikbke* (Figure 8E–J) all presented with an initial delay in activation but remained activated at the 2-week post-surgery time point. The delayed response of these six genes suggest that they could be potential co-therapeutic targets together with CD14, which displays a rapid response and can be targeted for microelectrode performance at acute time points [42]. Like many genes of the pattern recognition pathway, co-therapeutics with CD14 can be given sequentially, targeting first CD14 and later one of the TLRs. *Nfkb1* (Figure 8K) expression demonstrated its own unique pattern within the TLR pathway. Specifically, gene expression was modestly elevated compared to control animals at all but the 24-h post-surgery time point. NFkb is a transcription factor encoded by *Nfkb1*. NFkb responds to immune activation signals and in turn regulate immune response. Although we expect *Nfkb1* activity to play a role in the neuroinflammatory response against intracortical microelectrodes, it would be more important to evaluate the activity of NFkb rather than to conclude *Nfkb1* role based on gene expression alone.

### 3.5. Cytokine Response

Cytokines are small soluble protein molecules (~8–26 kDa) produced as a signaling molecule to modulate the immune response against pathogens and injury. Several classes of cytokines include chemokines, interferons, colony stimulating factors, lymphokines, and interleukins, which can be further subdivided into many families. Some members of the complement cascade, such as C4a, also act as cytokines [77].

**Figure 9:**
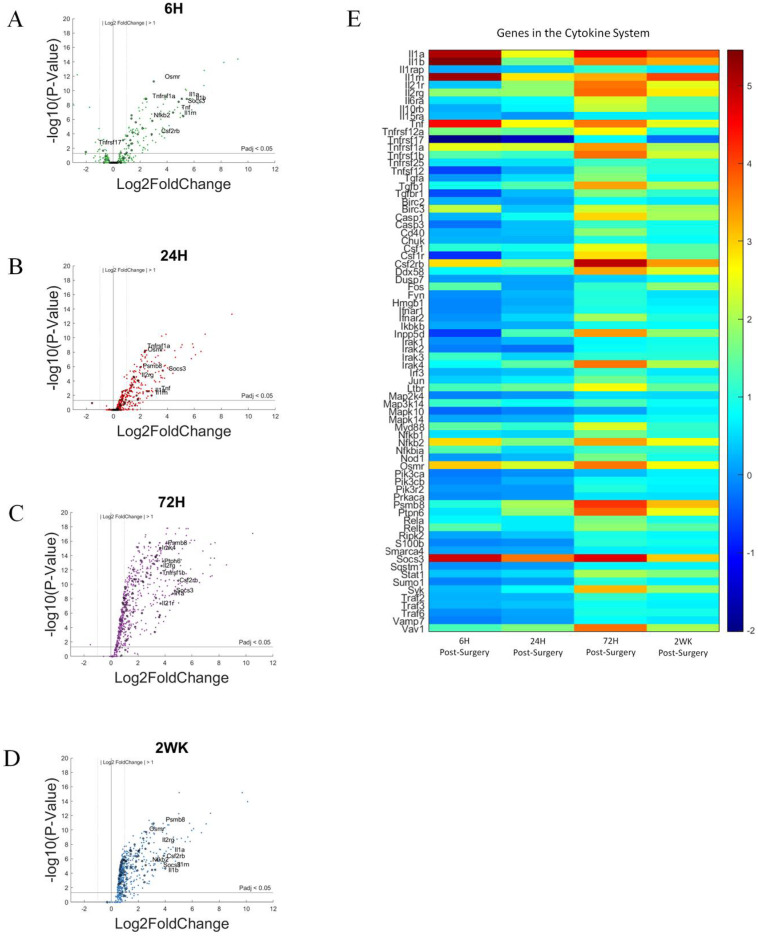
Differential expression of gene set involved in cytokine response compared to NSCTR mice: (**A**–**D**) volcano plot with genes in the cytokine system shown in black. Top 10 genes by differential expression level and P_adj_ < 0.05 are labeled. Each time point post-surgery is on a separate volcano plot. (**A**) =6-h, (**B**) =24-h, (**C**) =72-h, and (**D**) =2-weeks. Color in (**A**–**D**) corresponds to time post-surgery color coding in other figures. (**E**) heatmap showing differential expressions of genes of the cytokine system at each time point post-surgery compared to NSCTR.

Roughly 86 genes associated with the cytokine response were included in our panel. The 72 cytokine-associated genes that showed the largest differential gene expression in our study were compiled here for analysis and discussion. Figure 9 presents results for all genes in volcano plots, highlighting cytokine associated genes (Figure 9A–D). Additionally, we used a heat map to present log2foldchanges in gene expression for each time point examined, compared to NSCTR mice (Figure 9E).

In the volcano plots (Figure 9A–D), cytokine-associated genes are labeled if P_adj_ < 0.05 and log2foldchange > 1 or <−1 (i.e., 2-fold increase or decrease in expression), furthermore, due to the large number of gene in this grouping, only the top 10 genes with the largest log2foldchange at each time point within the group are labeled. All 72 cytokine associated genes are shown in Figure 9E which displays the relative gene expression levels of each time point compared to control in heatmaps.

**Figure 10:**
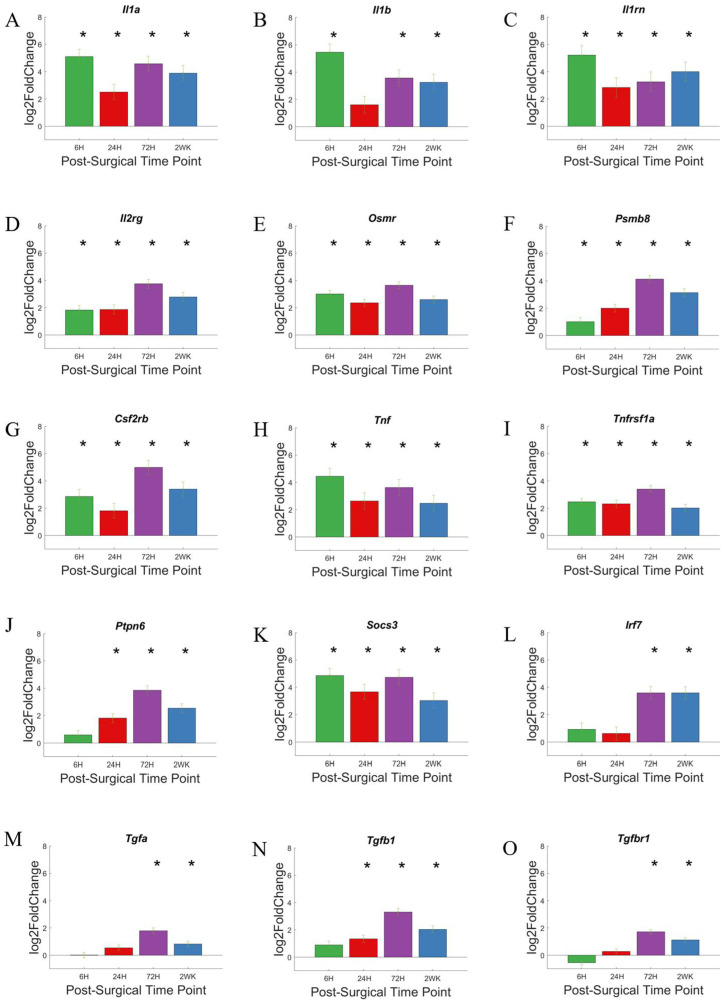
Differential expression of specific genes involved in the cytokine pathway compared to NSCTR mice: (**A**–**L**) Top differentially expressed genes for the cytokine gene set displayed as bar graphs of individual genes as a function of time post-surgery. For each time point, gene expression levels are compared to the NSCTR mice. (**M**–**O**) bar graph for TGF signaling pathways, which may be important for wound healing deemed important in the cytokine pathway. Error bars indicate the standard error of the mean between NSCTR and each time point. Asterisks indicate that P_adj_ < 0.05.

In Figure 10, we highlighted 12 cytokine-associated genes that were elevated for either 3 of the 4 times points we examined (*IL1b* and *Ptpn6*), or all 4 of the 4 times points we examined (*IL1a*, *IL1rn*, *IL2rg*, *Osmr*, *Psmb8*, *Csf2rb*, *Tnf*, *Tnfrsf1a*, *Socs3*, and *Vav1*), and 3 genes that showed elevation of 2 or 3 of the later time points that we examined (*Tgfa*, *Tgfb1*, and *Tgfbr1*). Each gene within this set shows a slightly different level of expression at each time point evaluated. Since we only ran statistical analysis between the time point and control mice, no statistical comparison will be made between individual time points, and only qualitative trends are warranted here.

Members of the chemokine family will be discussed in the next section. Members of Interleukin family of cytokines, such as *Il1a*, *Il1b*, *Il1rn*, and *Il2rg* (Figure 10A–D), and members of the Tumor Necrosis Factor (TNF) family, such as *Tnf* and *Tnfrs1a* (Figure 10H,I), showed increased expression at our earliest time point, with continued upregulation throughout the duration of this study. The Transforming Growth Factor (TGF) Family of genes, *Tgfa*, *Tgfb*, *Tgfbr1*, (Figure 10M–O) all showed a delay in increase in expression with a more pronounced increased in expression at the 72-h and/or 2-week time point.

Although cytokines do not directly interact with the microelectrode, cytokines do promote an inflammatory state in the tissue-microelectrode interface that may lead to a prolonged blood–brain barrier breakdown, production of damaging molecules such as reactive oxygen species, and reduced healing [78]. For example, *Tnfs* (Figure 10H,I) and *Ils* (Figure 10A–D) encodes pro-inflammatory molecules and rapid responders to injury [77].

Rapidly produced and accumulated high levels of cytokines reflect their role as key modulators and coordinators of the immune system. CD14 is an early detector of tissue damage and infection, and a lack of CD14 could potentially disrupt the gene-expression of cytokines. As cytokines form a complex and dynamic system of interactions, initial disruptions in expression of some of the cytokines may lead to altered inflammatory response at early time points post-implantation, and the system may recover at later time points. Members of the cytokine families may be great targets to improve recording performance, either alone or in combination with targeting CD14.

Gene encoding receptor for cytokine TGFβ, such as *Tgfbr1* (Figure 10O), showed no increase in expression until 72-h post-surgery and continue to show increased expression at 2-weeks post-surgery. The increase in expression later is consistent with the role of TGFβ as an anti-inflammatory molecule and its role in wound healing, which lags acute inflammation [79]. Due to its anti-inflammatory properties, TGFβ may not be a potential inhibitory therapeutic target in microelectrode implantation. However, TGFβ may represent be a biomarker to evaluate the inflammatory process in the tissue-microelectrode interface for research purposes.

### 3.6. Chemokines

Chemokines, or chemotactic cytokines, are a superfamily subgroup of cytokines. The main role of chemokines involves the promotion of migration of white blood cells to the site of injury or infection. Members of the chemokine superfamily are further divided into 4 families based on their protein structural motif: XC, CC, CXC, and CX3C [80,81]. Note: XC motif chemokines has one cysteine near its amino terminus, CC motif chemokines has two cysteine adjacent to each other, CXC motif chemokines has two cysteines separated by an amino acid in between, and CX3C motif chemokines has two cysteines separated by 3 amino acids in between.

**Figure 11:**
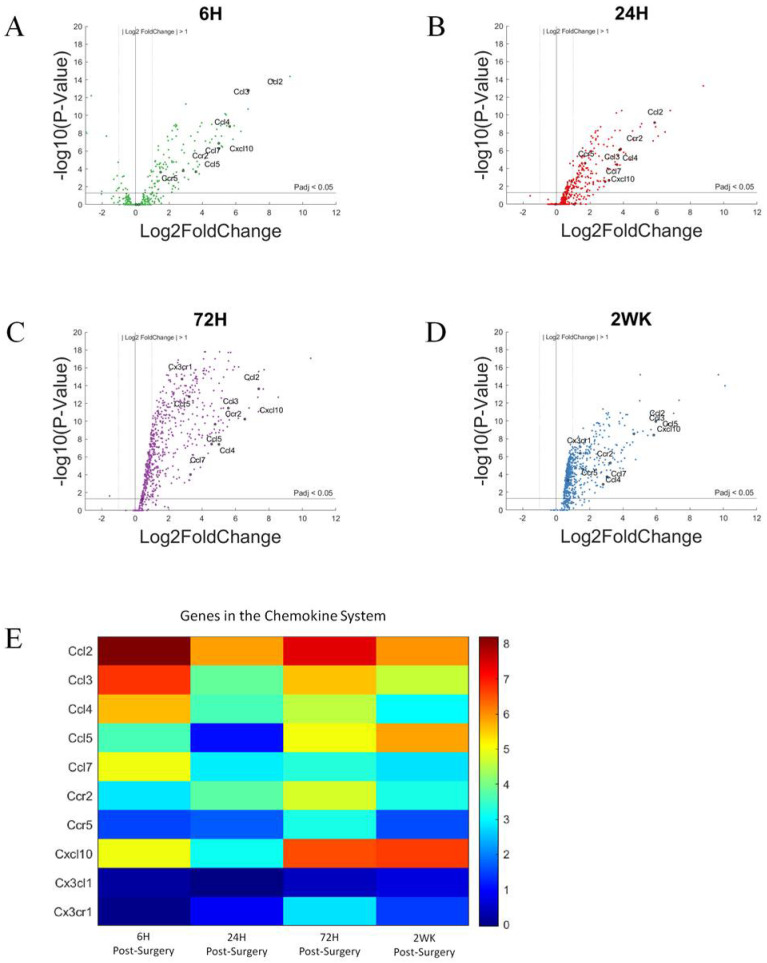
Differential expression of gene set involved in chemokine response compared to NSCTR mice: (**A**–**D**) volcano plot with genes in the chemokine system in black. Genes in the chemokine response system with P_adj_ < 0.05 and log2foldchange > 1 or <−1 are labeled. Each time point post-surgery is on a separate volcano plot. (**A**) =6-h, (**B**) =24-h, (**C**) =72-h, and (**D**) =2-weeks. Color in (**A**–**D**) corresponds to time post-surgery color coding in other figures. (**E**) heatmap showing differential expressions of genes of the chemokine system at each time point post-surgery compared to NSCTR.

**Figure 12:**
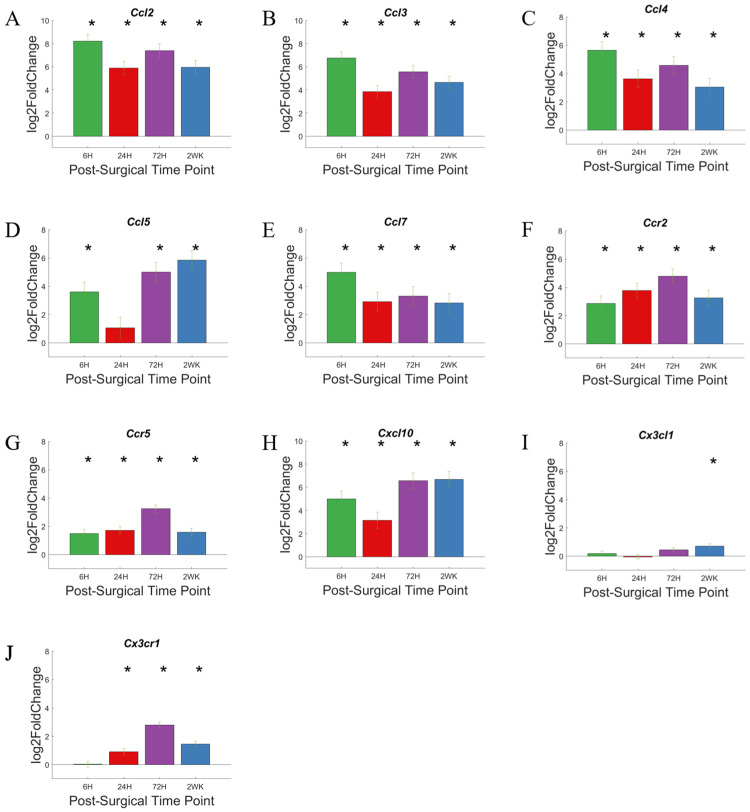
Differential expression of specific genes involved in the chemokine pathway compared to NSCTR mice: (**A**–**J**) Top differentially expressed genes for the chemokine gene set displayed as bar graphs of individual genes as a function of time post-surgery. For each time point, gene expression levels are compared to the NSCTR mice. Error bars indicate the standard error of the mean between NSCTR and each time point. Asterisks indicate that P_adj_ < 0.05. Note (**A**,**B**,**H**), which depicts the upregulation of *Ccl2*, *Ccl3*, and *Cxcl10*, respectively, has a y-axis log2foldchange scale of −1 to 10, because of their high upregulation.

Volcano plot presentation of changes of gene expression identified numerous chemokine associated genes that were upregulated following microelectrode implantation (Figure 11A–D). Specifically, the chemokines and associated genes: *Ccl2*, *Ccl3*, *Ccl4*, *Ccl5*, *Ccl7*, *Ccr2*, *Ccr5*, *Cxcl10*, *Cx3cl1*, and *Cx3cr1*, were labeled in Figure 11 if P_adj_ < 0.05 and log2foldchange > 1 or <−1 (i.e., 2-fold increase or decrease in expression). Chemokines display increased expression quickly after microelectrode implantation, with many of its members show upregulation in expression starting 6-h post-surgery and maintain high expression level throughout the 2-week period of this study. Specifically, at 6-h post-surgery, all chemokines studied excluding *Cx3cl1* and *Cx3ccr1* showed increased expression and remain elevated for the reminder of the 2-week study. However, *Cx3cr1* showed low levels of upregulation in expression at 72-h and 2-weeks post-surgery. The relative increase in gene expression levels for each of these genes for chemokine associated genes are more readily depicted in heatmaps (Figure 11E) and bar graphs (Figure 12). The latter also indicate statistical significance compared to non-surgical controls

Differential expression of individual genes (Figure 12A–J) plotted as bar graphs with distinction for significance versus the NSCTR mice allows us to note changes in activity versus time. Interestingly, most of the genes showing increased activity early on are of the CCL chemokine family (CC motif chemokine ligands): *Ccl2*, *Ccl3*, *Ccl4*, *Ccl5*, *Ccl7* (Figure 12A–E); and one member of the CXCL chemokine family (CXC motif chemokine ligands): *Cxcl10* (Figure 12H). After 6-h post-surgery, the genes are highly upregulated, at ~4–8-fold increase on log2 scale. In many cases the expression level of these genes remains high, although in Ccl5 the expression level decreases at 24-h post-surgery, just to recover to higher expression levels. Genes encoding receptors for the CCL family, the CC-Receptors *Ccr2* and *Ccr5* (Figure 12F,G), demonstrate a slow increase in expression with time, and are upregulated to a lesser degree than the CCL chemokine family.

Genes for the CX3C family ligand *Cx3cl1* and receptor *Cx2cr1* (Figure 12I,J) showed low levels of upregulation in expression level compared to the CC an CXC family of cytokines. *Cx3xl1* showed slight upregulation in gene expression, and while statistically significant only at 2-week post-surgery, with less than 1 log2foldchange. *Cx3cr1*(receptor for protein encoded by *Cx3cl*) showed a slightly higher upregulation in expression compared to its ligand, but still lower compared to genes encoding CC family of receptors.

In addition to recruiting cells of the immune system to the site of injury, chemokines are also involved in the proliferation, differentiation, activation, degranulation, and respiratory burst of white blood cells; their activities alter the microenvironment of the site of infection and injury. Respiratory burst, especially, leads to the production of reactive oxygen species that may damage implanted microelectrode as well as the tissue in the implant site. The CC subfamily of chemokines are involved in chemoattraction and induce the migration of immune cells such as monocytes [82]. The rapid and high upregulation of the CC chemokines suggest large numbers of monocytes would be recruited to the site of injury. The CXC subfamily of chemokines is also involved in the chemoattraction of immune cells such as neutrophiles [83]. The lone CX3C subfamily member of chemokines are involved in both chemoattraction and adhesion [84]. The high expression level of CCL and CXCL family present them as good potential targets in reducing inflammation and improving chronic microelectrode recording performance, either alone or in combination with CD14 inhibition.

### 3.7. Extracellular Matrix

The extracellular matrix (ECM) in the brain consists of insoluble proteins that forms a scaffold around the cells. The ECM helps to maintain the structural integrity of the tissue, mediate communication, stabilize synaptic contacts, and is important in neuroinflammation and wound healing [85].

**Figure 13:**
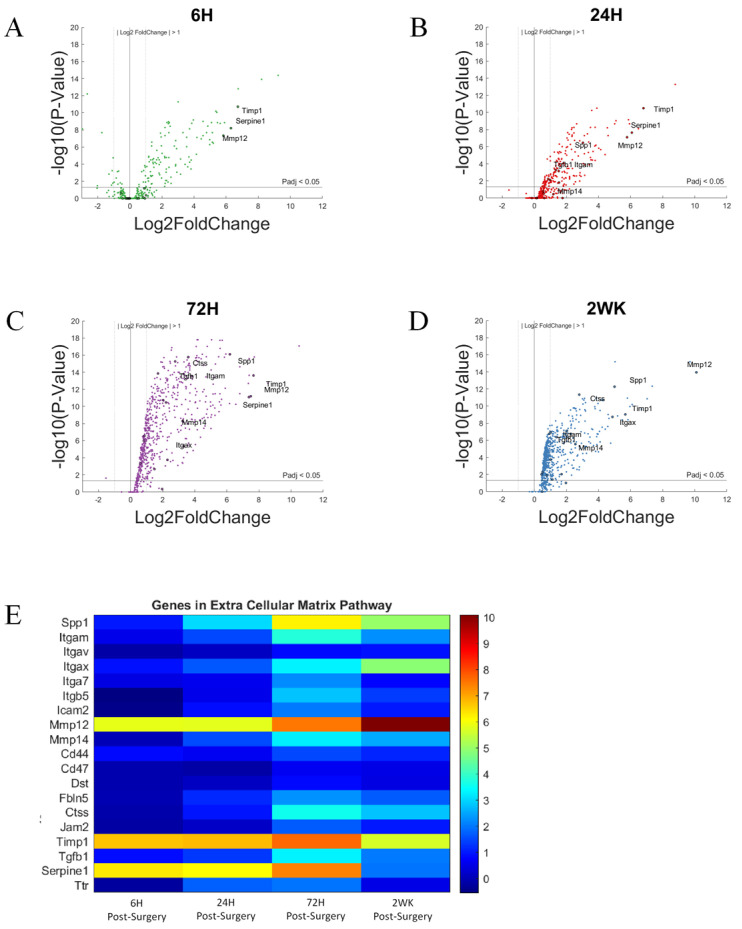
Differential expression of gene set involved in the extracellular matrix group compared to NSCTR mice: (**A**–**D**) volcano plot with genes in the extracellular matrix group in black. Top 10 genes by differential expression level and P_adj_ < 0.05. are labeled. Each time point post-surgery is on a separate volcano plot. (**A**) =6-h, (**B**) =24-h, (**C**) =72-h, and (**D**) =2-weeks. Color in (**A**–**D**) corresponds to time post-surgery color coding in other figures. (**E**) heatmap showing differential expressions of genes of the extracellular matrix group at each time point post-surgery compared to NSCTR.

In Figure 13A–D, we labeled genes associated with ECM if P_adj_ < 0.05 and log2foldchange > 1 or <−1 (i.e., 2-fold increase or decrease in expression). Furthermore, due to the large number of gene in this grouping, only the top 10 genes with the largest log2foldchange at each time point within the group are labeled. Most of the genes in the ECM pathway did not show increased expression at 6-h post-surgery. The genes showing increased expression at 6-h were *Mmp12*, *Timp1*, and *Serpine1*, and they remain elevated for the 2-week study. Some genes became upregulated steadily over the course of the 2-week study; these genes include *Spp1* and *Itgax*. Other genes remain lowly expressed over the course of the study; these genes include cell surface adhesion molecules *Itga7*, *Itgav*, *Itgam*. The relative increase in gene expression levels for each of these genes are more readily depicted in heatmaps (Figure 13E).

**Figure 14:**
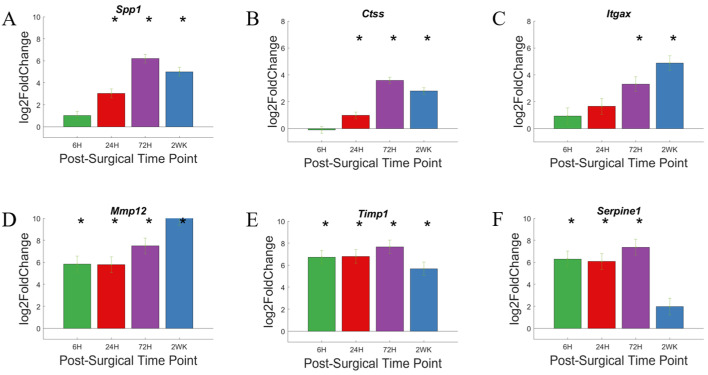
Differential expression of specific genes involved in the extracellular matrix pathway compared to NSCTR mice (**A**–**F**) Top differentially expressed genes for the extracellular matrix gene set displayed as bar graphs of individual genes as a function of time post-surgery. For each time point, gene expression levels are compared to the NSCTR mice. Error bars indicate the standard error of the mean between NSCTR and each time point. Asterisks indicate that P_adj_ < 0.05. Note (**A**,**D**,**E**,**F**) which depicts the upregulation of *Spp1*, *Mmp12*, *Timp1*, and *Serpine1*, respectively, has a y-axis log2foldchange scale of −1 to 10, because of their high upregulation.

Expression of individual genes at specific time points are depicted in bar graphs (Figure 14). *Spp1*, *Itgax*, and *Ctss* are genes that showed no significant upregulation at 6-h time point, and steadily increase their expression over the course of 2 weeks. *Spp1* and *Ctss* upregulation becomes significant by 24-h time point and reaches the maximum expression level by 72-h time point, before falling slightly by 2-week time point. *Itgax* expression level becomes significantly upregulated at 24-h time point and continue to increase over the 2 week study. A few genes, *Mmp12*, *Timp1*, and *serpine1*, showed rapid and high upregulation starting at 6-h post-surgery. *Mmp12* showed further upregulation in expression level over the course of the study. *Timp1* maintained an upregulation of gene expression until 72-h time point and begin to show a decrease in upregulation of gene expression by 2-week post-surgery. *Serpine1* maintained an upregulation of gene expression at 6–8 log2foldchange until 72-h time point and drops to below statistical significance by 2-weeks post-surgery.

The extracellular matrix in the central nervous system is produced by both neurons and glial cells and thought to occupy 20% of the volume of the brain. The structure of the ECM within the brain is unique: it consists of minimal collagen and fibronectin, and mainly consist of proteoglycans, glycoproteins, linker proteins, and matricellular proteins [85]. EMC undergoes constant modification during developmental and aging process, and the structure is thought to be heterogenous throughout the brain [86,87]. The brain’s ECM is thought to be involved in learning and memory [88,89], while alternations in ECM protein expression has been associated a variety of disorders such as Schizophrenia, Alzheimer’s, and epilepsy [90]. During injury and neural inflammation, ECM is actively remodeled to form scar tissue (in combination with astroglia scar) to prevent further damage to nearby neurons and promote recovery [91,92].

Matrix metalloproteases (MMPs) are zinc-containing endopeptidases involved in ECM maintenance. MMPs facilitate the breakdown and remodeling of extracellular matrix structural proteins and proteoglycans. The gene *Mmp12* codes for the protein matrix metalloproteases 12, which has been associated with injury and diseases such as stroke, spinal cord injury, and multiple scoliosis [93]. Minocycline, a non-specific MMP inhibitor that has demonstrated antibiotic and immune-modulating activities [94,95], has been shown to correlate with improved intracortical recording performances in rats provided minocycline in their drinking water for four weeks [96].

Tissue Inhibitor of Metalloproteases 1 (TIMP1) is an inhibitor of matrix metalloproteases, including MMP12. Therefore, *Timp1/Mmp12* ratio could be viewed as an indicator of proteolytic activity to the extracellular matrix [97,98]. Between 6-h and 72-h post-surgery, the *Timp1/Mmp12* ratio remains relatively steady: with both being upregulated. At 2-weeks post-surgery, the *Timp1* expression begin to decrease, while *Mmp12* expression keeps increasing. This may suggest a tip toward degradation and remodeling of extracellular matrix, an important step in wound healing.

*Serpine1* encodes for plasminogen activator inhibitor-1 (PAI-1) an inhibitor of tissue plasminogen activators (tPA) and urokinase plasminogen activators (uPA) [99]. tPA may generate plasmin, which may degrade laminin of the ECM as well as activate MMPs [100,101]. Hence, SERPINE1 may be considered a regulator of ECM remodeling. Decreased upregulation of *Serpine1* at 2-weeks post-surgery may indicated an increase in tPA activity, increased plasmin, and increased degradation and remodeling of extracellular matrix.

The continued upregulation of *Mmp12* over the course of the 2-week study and the upregulation of *Timp1* and *Serpine1* until 72-h time point and decline by 2-week time point, together, likely leads to an increased degradation and remodeling of the ECM. While ECM remodeling may affect the architecture of the tissue-microelectrode interface, leading to decreased recording quality of microelectrodes implanted in *Cd14*^−/−^ mice after the acute phase; it is more likely that the over-expression of degradative MMPs results in uncontrollable non-specific protein degradation which could impact membrane bound proteins in healthy neurons as well. Therefore, inhibition of MMPs may be a potential method to increase the recording performance at chronic time points, through increased cell viability. This hypothesis is still speculative and requires further investigation to confirm suspicions. Extracellular matrix remodeling is important for tissue integrity. Therefore, it may contribute to tissue architecture that reduces microelectrode performance. Thus, while not all the genes that encode for extracellular matrix proteins examined here show delayed response, when exploring genes of the extracellular matrix as potential therapeutic targets, we must consider the time course of inhibition, whether as a solo therapeutic target or as co-therapeutic targets with CD14.

## 4. Conclusions

The current study examined the expression of 791 genes in the neuroinflammatory pathway following microelectrode implantation into the cortex of *Cd14*^−/−^ mice. Gene expression for tissue within 500 µm of the microelectrode-tissue interface was analyzed. Previous studies have shown CD14 to be a potential therapeutic target in improving microelectrode recording performances over the same period described in this study. Here, our goal was to investigate the changes in expression of genes in the neuroinflammatory pathways in *Cd14*^−/−^ mice, detect gene-expression patterns that may confer its ability to improve microelectrode recording performance at acute time points, and identify potential therapeutic target that could be used in combination or succession of CD14 inhibition to improve the microelectrode performance.

We found that the greatest variation and the highest level of gene expression upregulation occurs at 72-h time point post-surgery, which coincides with the time of transition from a “inflammatory phase’ to a “healing phase” in tissue injury. Note that this is delayed compared to WT mice from a previous study, where the greatest variation and the highest level of gene expression upregulation occurs at 24-h time point post-surgery [36]. The time course of upregulation of gene expression may prove important for the dynamics of inflammation, which may hold the key to the initial and short-lived improvements of microelectrode performance in *Cd14*^−/−^ animals.

Previous studies in our lab have shown that partial inhibition of CD14 had improved microelectrode performance and is a potential therapeutic target. The current study strengthens our understanding of the molecular level tissue response to microelectrode implant over the first two weeks post-surgery in *Cd14*^−/−^ animals, over the same duration in which *Cd14* inhibition improved microelectrode performance. We have found the genes of the complement and chemokine system to be highly and rapidly upregulated, while genes in the cytokine system (non-chemokine), pattern recognition receptors, and Toll-like receptors to be less upregulated. Genes in the extracellular matrix system consist of a few highly upregulated proteolytic enzymes and their inhibitors. Rapidly and highly upregulated genes, such as *C3* of the complement system, *CXCL10* of the chemokine system, and *Mincle* of the pattern recognition system are potential therapeutic target in improving microelectrode performance, either alone or in combination with *Cd14*. Genes showing delayed upregulation such as *Aim2* of pattern recognition pathway, *Itgax* which is involved in extracellular matrix remodeling, can be potential co-therapeutic targets that may be targeted with *Cd14* sequentially. The suggestions for targets provided here will require further validation of protein expression levels to determine the best means to attenuate or silence gene and proteins of importance.

One limitation of this study is that we did not look at protein expression. Gene expression is a proxy for protein expression, which are the machinery that controls tissue response. Another limitation is the lack of precise spatial resolution. We expect the largest changes in gene expression to be closer to implant site, while for this study we pooled together all gene expression within 500 µm of the implant site based on the methods available to us at the onset of the study. Future studies should investigate both the gene and protein expression on a cell-specific, spatially defined level with increased resolution, like that offered in the NanoString GeoMx platform, while also utilizing functional microelectrode arrays to assess device performance.

## Figures and Tables

**Table 1:** A comprehensive list of genes investigated in this study.

Abcc3	Bnip3l	Cdc7	Dock2	Gfap	Il1rl2	Lcn2	Myc	Plcg2	Ripk1	Spib	Topbp1
Abcc8	Bok	Cdk20	Dot1l	Gja1	Il1rn	Ldha	Myct1	Pld1	Ripk2	Spint1	Tpd52
Abl1	Bola2	Cdkn1a	Dst	Gjb1	Il21r	Ldlrad3	Myd88	Pld2	Rnf8	Spp1	Tpsb2
Adamts16	Braf	Cdkn1c	Duoxa1	Gna15	Il2rg	Lfng	Myrf	Plekhb1	Rpa1	Sqstm1	Tradd
Ago4	Brca1	Ceacam3	Dusp7	Gpr183	Il3	Lgmn	Nbn	Plekhm1	Rpl28	Srgn	Traf1
Agt	Brd2	Cflar	E2f1	Gpr34	Il36ra	Lig1	Ncaph	Pllp	Rpl29	Srxn1	Traf2
AI464131	Brd3	Ch25h	Eed	Gpr62	Il3ra	Lilrb4a	Ncf1	Plp1	Rpl36al	St3gal6	Traf3
Aim2	Brd4	Chek1	Eef2k	Gpr84	Il6ra	Lingo1	Ncor1	Plxdc2	Rpl9	St8sia6	Traf6
Ak1	Btk	Chek2	Egfr	Grap	iNos	Lmna	Ncor2	Plxnb3	Rps10	Stat1	Trat1
Akt1	C1qa	Chn2	Egr1	Gria1	Inpp5d	Lmnb1	Ncr1	Pmp22	Rps2	Steap4	Trem1
Akt2	C1qb	Chst8	Ehd2	Gria2	Iqsec1	Lrg1	Nefl	Pms2	Rps21	Stmn1	Trem2
Aldh1l1	C1qc	Chuk	Ehmt2	Gria4	Irak1	Lrrc25	Nfe2l2	Pnoc	Rps3	Stx18	Trem3
Ambra1	C3	Cidea	Eif1	Grin2a	Irak2	Lrrc3	Nfkb1	Pole	Rps9	Sumo1	Trim47
Amigo2	C3ar1	Cideb	Emcn	Grin2b	Irak3	Lsr	Nfkb2	Ppfia4	Rrm2	Suv39h1	Trp53
Anapc15	C4a	Cks1b	Emp1	Grm2	Irak4	Lst1	Nfkbia	Ppp3ca	Rsad2	Suv39h2	Trp53bp2
Anxa1	C5ar1	Clcf1	eNos	Grm3	Irf1	Lta	Nfkbie	Ppp3cb	Rtn4rl1	Suz12	Trp73
Apc	C6	Cldn5	Enpp6	Grn	Irf2	Ltb	Ngf	Ppp3r1	S100a10	Syk	Trpa1
Apex1	Cables1	Clec7a	Entpd2	Gsn	Irf3	Ltbr	Ngfr	Ppp3r2	S100b	Syn2	Trpm4
Apoe	Calcoco2	Clic4	Eomes	Gstm1	Irf4	Ltc4s	Ninj2	Prdx1	S1pr3	Syp	Tspan18
App	Calr	Cln3	Ep300	Gzma	Irf6	Ly6a	Nkg7	Prf1	S1pr4	Tarbp2	Ttr
Aqp4	Camk4	Clstn1	Epcam	Gzmb	Irf7	Ly6g	Nlgn1	Prkaca	S1pr5	Tbc1d4	Tubb3
Arc	Casp1	Cnn2	Epg5	H2afx	Irf8	Ly9	Nlgn2	Prkacb	Sall1	Tbr1	Tubb4a
Arg1	Casp2	Cnp	Epsti1	H2-T23	Islr2	Lyn	Nlrp2	Prkar1a	Scd1	Tbx21	Txnrd1
Arhgap24	Casp3	Cntnap2	Erbb3	Hat1	Itga6	Mafb	Nlrp3	Prkar2a	Sell	Tcirg1	Tyrobp
Arid1a	Casp4	Coa5	Ercc2	Hcar2	Itga7	Maff	nNos	Prkar2b	Serpina3n	Tcl1	Ugt8a
Asb2	Casp6	Col6a3	Ercc6	Hdac1	Itgam	Mag	Nod1	Prkce	Serpine1	Tet1	Ulk1
Ash2l	Casp7	Cotl1	Esam	Hdac2	Itgav	Mal	Nostrin	Prkcq	Serpinf1	Tfg	Ung
Asph	Casp8	Cox5b	Ets2	Hdac4	Itgax	Man2b1	Noxa1	Prkdc	Serping1	Tgfa	Uty
Atf3	Casp9	Cp	Exo1	Hdac6	Itgb5	Map1lc3a	Npl	Prnp	Sesn1	Tgfb1	Vamp7
Atg14	Cass4	Cpa3	Ezh1	Hdc	Jag1	Map2k1	Npnt	Pros1	Sesn2	Tgfbr1	Vav1
Atg3	Ccl2	Creb1	Ezh2	Hells	Jam2	Map2k4	Nptx1	Psen2	Setd1a	Tgm1	Vegfa
Atg5	Ccl3	Crebbp	F3	Hif1a	Jarid2	Map3k1	Nqo1	Psmb8	Setd1b	Tgm2	Vim
Atg7	Ccl4	Crem	Fa2h	Hilpda	Jun	Map3k14	Nrgn	Pten	Setd2	Tie1	Vps4a
Atg9a	Ccl5	Crip1	Fabp5	Hira	Kat2a	Mapk10	Nrm	Ptger3	Setd7	Timeless	Vps4b
Atm	Ccl7	Cryba4	Fadd	Hist1h1d	Kat2b	Mapk12	Nrp2	Ptger4	Setdb1	Timp1	Was
Atp6v0e	Ccng2	Csf1	Fancc	Hmgb1	Kcnd1	Mapk14	Nthl1	Ptgs2	Sftpd	Tle3	Wdr5
Atp6v1a	Ccni	Csf1r	Fancd2	Hmox1	Kcnj10	Mapt	Nwd1	Ptms	Sh2d1a	Tlr2	Xcl1
Atr	Ccr2	Csf2rb	Fancg	Homer1	Kcnk13	Marco	Oas1g	Ptpn6	Shank3	Tlr4	Xiap
Axl	Ccr5	Csf3r	Fas	Hpgds	Kdm1a	Mavs	Ogg1	Ptprc	Siglec1	Tlr7	Xrcc6
B3gnt5	Cd109	Csk	Fasl	Hprt	Kdm1b	Mb21d1	Olfml3	Pttg1	Siglecf	Tm4sf1	Zbp1
Bad	Cd14	Cst7	Fbln5	Hps4	Kdm2a	Mbd2	Opalin	Ptx3	Sin3a	Tmc7	Zfp367
Bag3	Cd163	Ctse	Fcer1g	Hrk	Kdm2b	Mbd3	Optn	Pycard	Sirt1	Tmcc3	Aars
Bag4	Cd19	Ctsf	Fcgr1	Hsd11b1	Kdm3a	Mcm2	Osgin1	Rab6b	Slamf8	Tmem100	Asb10
Bak1	Cd209e	Ctss	Fcgr2b	Hspb1	Kdm3b	Mcm5	Osmr	Rab7	Slamf9	Tmem119	Ccdc127
Bard1	Cd244	Ctsw	Fcgr3	Hus1	Kdm4a	Mcm6	P2rx7	Rac1	Slc10a6	Tmem144	Cnot10
Bax	Cd24a	Cx3cl1	Fcrla	Icam2	Kdm4b	Mdc1	P2ry12	Rac2	Slc17a6	Tmem173	Csnk2a2
Bbc3	Cd300lf	Cx3cr1	Fcrlb	Ifi30	Kdm4c	Mdm2	Pacsin1	Rad1	Slc17a7	Tmem204	Fam104a
Bcas1	Cd33	Cxcl10	Fcrls	Ifih1	Kdm4d	Mef2c	Padi2	Rad17	Slc1a3	Tmem206	Gusb
Bcl10	Cd36	Cxcl9	Fdxr	Ifitm2	Kdm5a	Mertk	Pak1	Rad50	Slc2a1	Tmem37	Lars
Bcl2	Cd3d	Cycs	Fen1	Ifitm3	Kdm5b	Mfge8	Parp1	Rad51	Slc2a5	Tmem64	Mto1
Bcl2a1a	Cd3e	Cyp27a1	Fgd2	Ifnar1	Kdm5c	Mgmt	Parp2	Rad51b	Slc44a1	Tmem88b	Supt7l
Bcl2l1	Cd3g	Cyp7b1	Fgf13	Ifnar2	Kdm5d	Mincle	Pcna	Rad51c	Slc6a1	Tnf	Tada2b
Bcl2l11	Cd40	Cytip	Fgl2	Igf1	Kdm6a	Mmp12	Pdpn	Rad9a	Slco2b1	Tnfrsf10b	Tbp
Bcl2l2	Cd44	Dab2	Fkbp5	Igf1r	Kif2c	Mmp14	Pecam1	Rag1	Slfn8	Tnfrsf11b	Xpnpep1
Bdnf	Cd47	Dapk1	Flt1	Igf2r	Kir3dl1	Mobp	Pex14	Rage	Smarca4	Tnfrsf12a	
Becn1	Cd6	Ddb2	Fos	Igsf10	Kir3dl2	Mog	Pik3ca	Rala	Smarca5	Tnfrsf13c	
Bid	Cd68	Ddx58	Foxp3	Igsf6	Kit	Mpeg1	Pik3cb	Ralb	Smarcd1	Tnfrsf17	
Bik	Cd69	Dicer1	Fpr1	Ikbkb	Klrb1	Mpg	Pik3cd	Rapgef3	Smc1a	Tnfrsf1a	
Bin1	Cd70	Dlg1	Fscn1	Ikbke	Klrd1	Mr1	Pik3cg	Rb1cc1	Snca	Tnfrsf1b	
Birc2	Cd72	Dlg4	Fyn	Ikbkg	Klrk1	Mre11a	Pik3r1	Rbfox3	Socs3	Tnfrsf25	
Birc3	Cd74	Dlx1	Gadd45a	Il10rb	Kmt2a	Ms4a1	Pik3r2	Rela	Sod1	Tnfrsf4	
Birc5	Cd83	Dlx2	Gadd45g	Il15ra	Kmt2c	Ms4a2	Pik3r5	Relb	Sod2	Tnfsf10	
Blk	Cd84	Dna2	Gal3st1	Il1a	Lacc1	Ms4a4a	Pilra	Reln	Sod3	Tnfsf12	
Blm	Cd86	Dnmt1	Gba	Il1b	Lag3	Msh2	Pilrb1	Reserved	Sox10	Tnfsf13b	
Blnk	Cd8a	Dnmt3a	Gbp2	Il1r1	Lair1	Msn	Pink1	Rgl1	Sox4	Tnfsf4	
Bmi1	Cd8b1	Dnmt3b	Gclc	Il1r2	Lamp1	Msr1	Pla2g4a	Rhoa	Sox9	Tnfsf8	
Bnip3	Cdc25a	Dock1	Gdpd2	Il1rap	Lamp2	Mvp	Pla2g5	Rig1	Sphk1	Top2a	

List of genes investigated in the study. A total of 791 genes are listed (6 control sequences are excluded in the analysis): genes from the nCounter^®^ Mouse Neuroinflammation Panel are in **black,** 20 custom genes of interest are in **blue**, and housekeeping genes are in **red**.

## Data Availability

Please send request to jrc35@case.edu.
